# Effect of Inulin and Psyllium Husk Powder on Gel Properties and In Vitro Digestion of *Hypophthalmichthys molitrix* and *Argopecten irradians* Blended Surimi

**DOI:** 10.3390/foods13223703

**Published:** 2024-11-20

**Authors:** Wenhao Geng, Miaomiao Tian, Xinyue Zhang, Maodong Song, Xinru Fan, Meng Li, Yongsheng Ma, Soottawat Benjakul, Qiancheng Zhao

**Affiliations:** 1 College of Food Science and Engineering, Dalian Ocean University, Dalian 116023, China; 2 Dalian Key Laboratory of Marine Bioactive Substances Development and High Value Utilization, Dalian 116023, China; 3 Liaoning Provincial Marine Healthy Food Engineering Research Centre, Dalian 116023, China; 4 International Center of Excellence in Seafood Science and Innovation, Faculty of Agro-Industry, Prince of Songkla University, Hat Yai 90110, Songkhla, Thailand; soottawat.b@psu.ac.th

**Keywords:** psyllium husk powder (PHP), inulin (INU), mixed surimi, textural properties, in vitro digestion

## Abstract

Dietary fiber is crucial in enhancing the nutritional and textural properties of surimi-based products. This study investigated blended surimi produced from silver carp and bay scallops, with the addition of different amounts (0%, 0.5%, 1%, 2%, and 3%) of inulin (INU) or psyllium husk powder (PHP) for their textural properties, protein conformation, and in vitro digestibility. The addition of INU negatively affected gel strength. However, incorporating 2.0% PHP into the blended gel improved gel strength and water-holding capacity by 8.01% and 0.79% compared to the control, respectively. Furthermore, PHP significantly increased the total sulfhydryl content and surface hydrophobicity of the blended gels (*p* < 0.05). Additionally, increases in endogenous fluorescence intensity accompanied by a blue shift were observed, indicating that the fluorophores (Trp and Tyr) were sequestered into a more non-polar environment due to conformational changes. The incorporation of PHP enhanced both the quality and digestibility of the blended surimi. This study provides a novel perspective for developing surimi-based food with improved quality, augmented digestion, and enhanced absorption.

## 1. Introduction

Nowadays, individuals increasingly strive for a healthy lifestyle. Poor dietary choices can have various long-term adverse effects and elevate the risk of problems such as cancer and cardiovascular diseases. Developing products rich in protein with low fat, reduced sodium, and minimal additives has emerged as a potential approach to health-promoting food [1]. Surimi is recognized as a high-protein and low-fat processed aquatic product without religious restrictions. Freshwater fish, particularly silver carp (*Hypophthalmichthys molitrix*), are promising raw materials for surimi production due to their rapid growth rate, abundant yield, and cost-effectiveness [2]. To enhance the textural property and flavor profile, crustacean and cephalopod meat have been blended with freshwater fish surimi. Incorporating scallop mince at an appropriate proportion in silver carp surimi has yielded high-quality gel with favorable sensorial characteristics [3]. However, surimi-based products lack dietary fiber, causing a nutritional imbalance. Safe and nutritious exogenous additives have been incorporated to overcome this issue while retaining or improving the textural properties of surimi gels.

Certain dietary fibers exhibit antioxidant properties and possess the capacity to retain water and oil, as well as the ability to adsorb cholesterol. Moreover, they can modify food texture depending on the type and quantity of fiber utilized [4]. Incorporating dietary fiber from diverse sources into surimi-based products has been extensively investigated [4]. Inulin (INU) is a linear polydisperse carbohydrate primarily composed of D-fructose linked by beta-(2→1) bonds [5]. It promotes the growth of beneficial gut microbiota, reduces blood sugar levels, enhances mineral absorption, and bolsters immune function [6]. Inulin with varying chain lengths can maintain a stable protein structure while entrapping water molecules within surimi, improving water retention and freeze–thaw stability [7,8]. Psyllium husk powder (PHP), obtained by grinding psyllium seed husks, is recognized for its cost-effectiveness, straightforward processing methods, and health benefits, such as intestinal hydration, constipation relief, blood sugar reduction, and increased satiety [9]. PHP has been verified to enhance cross-linking and aggregation with protein molecules, resulting in a denser gel network from *Andrias davidianus* myofibrillar protein (MP) and improved water retention [10].

The structural characteristics of food significantly influence its digestibility. Surimi is an exemplary food substrate; it is rich in high-quality protein, and the gel properties of proteins are highly relevant to their digestibility [11]. Moreover, the nutritional value of protein is primarily determined by its digestibility within the gastrointestinal tract, which subsequently affects the bioavailability of amino acids and impacts overall amino acid metabolism [12,13]. The effects of adding dietary fiber on the gel properties of surimi, along with its potential positive effects on gastrointestinal digestion and absorption, warrant further investigation. Therefore, this study aimed to modify the properties of blended surimi gels derived from silver carp and bay scallops (*Argopecten irradians*) by incorporating INU and PHP. Furthermore, the impact of dietary fiber on the digestion and absorption of blended gels was investigated. This study supplies innovative insights for developing novel surimi-based products, particularly in supporting tailored diets for individuals with specific health conditions.

## 2. Materials and Methods

### 2.1. Materials

Fresh silver carp (H. *molitrix*, *n* = 20, weight = 1.5–2.0 kg) were supplied from a local market, packed in crushed ice (Dalian, Liaoning Province, China), and delivered to the lab within 30 min. Frozen scallops (A. *irradians* adductor muscle (5 kg)) were bought from the Dalian Yuyang Group Co. Ltd. (Dalian, China). INU (food grade) and PHP (food grade) were acquired from Wuhan Guanying Biotechnology Co., Ltd. (Wuhan, China). All chemical reagents were analytical grade (Shanghai Maclin Biochemical Technology Co., Ltd., Shanghai, China).

### 2.2. Sample Preparation

All silver carp were killed instantly with a sharp blow to the head by the vendor. The slaughtered fish samples were scaled, skinned, gutted, washed with iced water, and filleted within 1 h. The fish mince was collected without bones and ground with a grinder (QSJ-C03T5, Bear Electric Appliance Co., Ltd., Foshan, China) for 5 min at less than 10 °C. The scallop samples were thawed overnight at 4 °C and minced as described above. Based on our preliminary study [3], the scallop and silver carp mince were blended at the a 1:3 ratio with 2.5% NaCl (*w*/*w*). Subsequently, INU and PHP were directly added to the paste at varying concentrations (0.5%, 1.0%, 2.0%, and 3.0%, *w*/*w*) and mixed for 5 min. Finally, the moisture content was adjusted to 78% for all the samples. The mixture was packed into a cylindrical plastic casing (diameter = 25 mm, height = 30 mm) and incubated at 90 °C for 30 min. All samples were instantly chilled in iced water for 10 min and stored overnight in a refrigerator for follow-up measurements. The samples were pre-equilibrated at room temperature (RT, 25 °C) for 30 min before subsequent measurements.

### 2.3. Texture Profile Analysis (TPA) and Gel Strength

A TMS-PRO texture analyzer (Food Technology Corporation, Sterling, VA, USA) equipped with a P/25 probe was used for TPA implementation [14]. Testing conditions were as follows: a test speed at of 1.0 mm/s, compressive strain of 50%, and trigger force at 5.0 g. The gel strength of the samples was measured by the same instrument using a spherical probe (P/5S) with a test speed of 1 mm/s and a strain of 50% [15]. Pre-equilibrated samples were measured with six repetitions.

### 2.4. Water-Holding Capacity (WHC)

In brief, the gel samples (0.5 g, W_1_) were covered with tissue paper. After centrifugation (6873× *g*, 4 °C, 5 min), the weight of the gel was recorded as W_2_. The percentage of W_2_ relative to W_1_ is reported as WHC [16].

### 2.5. Protein Solubility of Blended Gel

Briefly, the gel samples were dissolved in 20 volumes of buffer (20 mmol/L Tris-HCl (pH 6.8), 2% SDS, 8 mol/L urea, 5% β-mercaptoethanol) based on the method of Benjakul and Visessanguan [17]. The supernatant of the mixture was collected via centrifugation (13,300× *g* for 15 min). Subsequently, 10 volumes of 7.5% trichloroacetic acid (TCA) were mixed with the supernatant (4 °C, 24 h) to prepare the precipitate (1718× *g* for 10 min). The sediment was rinsed with the same TCA solution and redissolved in 0.5 mol/L NaOH solution. The protein concentration was estimated using the Biuret method.

### 2.6. Color Analysis

L*, a*, and b*-values of blended gels were determined by a colorimeter (Konica Minolta, CR-400, Tokyo, Japan). The whiteness was calculated according to the method of Mi, et al. [18].

### 2.7. Scanning Electron Microscopy (SEM)

The gel cubes (10 × 5 × 5 mm^3^) were prepared via lyophilization. The cross-sections of the dried samples were observed using a JSM-7800F scanning electron microscope (Japan Electron Optics Laboratory, Tokyo, Japan) [19].

### 2.8. In Vitro Digestion Analysis

#### 2.8.1. In Vitro Digestion

An in vitro digestion system that included simulated salivary fluid (SSF), gastric fluid (SGF), and intestinal fluid (SIF) was employed [20]. In the oral phase, the blended gel (5.0 g) was ground and mixed with 3.5 mL of SSF (0.5 mol/L KCl, 0.5 mol/L KH_2_PO_4,_ 1 mol/L NaHCO_3_, 0.15 mol/L MgCl_2_·6 (H_2_O), and 0.5 mol/L (NH_4_)_2_CO_3_). Subsequently, 0.5 mL of 1500 U/mL salivary α-amylase solution, 25 μL of 0.3 mol/L CaCl_2_, and 975 μL of distilled water were added and incubated at 37 °C (200 rpm for 5 s). For the gastric phase, the above oral mixture was blended with 7.5 mL SGF (0.5 mol/L KCl, 0.5 mol/L KH_2_PO_4_, 1 mol/L NaHCO_3_, 2 mol/L NaCl, 0.15 mol/L MgCl_2_·6 (H_2_O), and 0.5 mol/L (NH_4_)_2_CO_3_), 1.6 mL porcine pepsin stock solution (2000 U/mL), 5 μL of 0.3 mol/L CaCl_2_, and 695 μL of distilled water. Subsequently, 0.2 mL of 6 mol/L of HCl was used to adjust the pH to 3.0. The digestive reaction was monitored at 0, 5, 15, 30, 45, 60, 90, and 120 min. Samples were held at 37 °C and 200 rpm using a stable temperature horizontal shaking bath (Shanghai Longyue Instrument Equipment Co., Ltd., Shanghai, China). In the intestinal phase, gastric chyme (20 mL) was mixed with 11.0 mL of SIF (0.5 mol/L KCl, 0.5 mol/L KH_2_PO_4_, 1 mol/L NaHCO_3_, 2 mol/L NaCl, 0.15 mol/L MgCl_2_·6 (H_2_O), 5.0 mL of trypsin (100 U/mL), 2.5 mL of 160 mmol/L fresh bile, and 40 μL of 0.3 mol/L CaCl_2_. The mixture was adjusted to pH 7.0 using 0.15 mL of 1 mol/L NaOH. The reaction was performed at 37 °C (200 rpm), and samples were taken at 0, 30, 60, 90, 120, 150, and 180 min for analysis.

#### 2.8.2. Protein Digestibility (PD)

The digestion samples were centrifuged at 6873× *g* for 20 min at 4 °C. The precipitate of the digestive samples was collected and weighed [1]. The protein digestibility was computed as follows:

$$PD\%=\left(1-\frac{m_{1}}{m_{0}}\right)\times 100\%$$

where *m*_0_ is the protein content of the original blended gel (g) before digestion, and *m*_1_ is the protein content of the precipitate (g) after digestion.

#### 2.8.3. Appearance and Optical Microscopy of Digestive Products

The visual appearance of the digesta was photographed after gastric and intestinal digestion. The microstructural images of digestive products were stained using 0.1% toluidine blue and studied with light microscopy (Leica DM 3000, Wetzlar, Germany).

#### 2.8.4. Particle Size and Zeta Potential of Digestive Products

The protein concentration of the digesta was diluted to 0.5 mg/mL with 20 mmol/L phosphatic buffer (Na_2_HPO_4_–NaH_2_PO_4_, pH 7.0). At RT, the particle size and zeta potential were measured using a Zetasizer Pro Analyzer (Malvern Instruments Ltd., Worcestershire, UK).

#### 2.8.5. Total Sulfhydryl Content of Digestive Products

The total sulfhydryl content (TSC) of the digestive products was determined by the method of digestive products [21]. The samples were mixed with 9 volumes of Tris-HCl buffer (0.2 mol/L Tris, 8 mol/L carbamide, 0.2 g/kg sodium dodecyl sulfate, and 10 mmol/L ethylenediaminetetraacetic acid, pH 6.8) and the same volume of 10 mmol/L 5,5′-dithiobis-(2-nitrobenzoic acid). The reaction was incubated at 40 °C for 25 min, and the absorbance at 412 nm was measured.

#### 2.8.6. Surface Hydrophobicity of Digestive Products

The diluted digestive products (5.0 mL) at concentrations ranging from 0.1 to 0.5 mg/mL were mixed with 25 μL of 8-anilino-1-naphthalenesulfonic acid ammonium salt (ANS, 10 mmol/L, pH 7.5) for 20 min (dark condition, 4 °C) [22]. The fluorescence intensity of samples was determined by a microplate reader (Synergy H1/H1M, BioTek Instruments, Winooski, VT, USA) at an excitation wavelength of 375 nm and an emission wavelength of 485 nm. The slope of the fluorescence intensity and protein concentration curve was defined as ANS-S_0_.

#### 2.8.7. Endogenous Fluorescence Spectrum for Digestive Products

The endogenous fluorescence emission spectra of the digestive products (0.5 mg/mL) were monitored following the method of Niu, et al. [23], with an excitation wavelength of 280 nm (scan range from 220 to 450 nm), slit widths of 5 nm, a scan velocity of 1500 nm/min, and a data collection interval of 1 nm^−1^.

### 2.9. Statistical Analysis

All experiments were conducted independently in three batches with triplicates. SPSS Statistics 25 version (SPSS Inc., Chicago, IL, USA) was used for analysis, and one-way ANOVA (Duncan’s test) was used for mean comparison at a significance level of *p* < 0.05. Diagrams were plotted using Origin 2021 (OriginLab Co., Northampton, MA, USA).

## 3. Result and Discussion

### 3.1. TPA of Blended Gels with Varying Levels of Added Dietary Fibers

The quality of surimi-based products is contingent upon their textural characteristics, which significantly influence consumer acceptance and preferences. TPA has been employed to simulate oral mastication via double compression, thereby exploring the textural properties of the gels [24]. The effects of dietary fibers on the textural properties of blended gels are presented in Table 1. Notably, a decline in texture strength was observed as the amount of INU increased. Compared with the control group, incorporating 3% INU reduced the hardness, cohesiveness, springiness, and chewiness by 9.84%, 22.00%, 5.54%, and 32.76%, respectively. The impact of INU on the gel texture was influenced by the degree of polymerization and the quantity added. Short-chain INU (degree of polymerization: 2–6) at a concentration of 4% enhanced gel hardness, cohesiveness, and chewiness compared to the control [7]. Conversely, the maximum hardness (56.43 ± 4.75 N), springiness (5.68 ± 0.34), and chewiness (146.71 ± 8.9 mJ) were achieved when the PHP concentration was 2% (*p* < 0.05). Consistent with previous findings Zhou, et al. [25], adding 2% PHP was optimal for gelatinous modification. This phenomenon may be attributed to hydrogen bonding and electrostatic interactions between the PHP and the protein molecules within the gel network that enhanced the anti-deformation capabilities and overall gel formation [26]. However, higher concentrations of PHP reduced hardness and chewiness due to excessive PHP filling the gel network, which disrupted cross-linking among the protein chains while decreasing the chemical interactions between the protein molecules, compromising the textural properties of the gels [25].

### 3.2. Gel Strength

**Figure 1:**
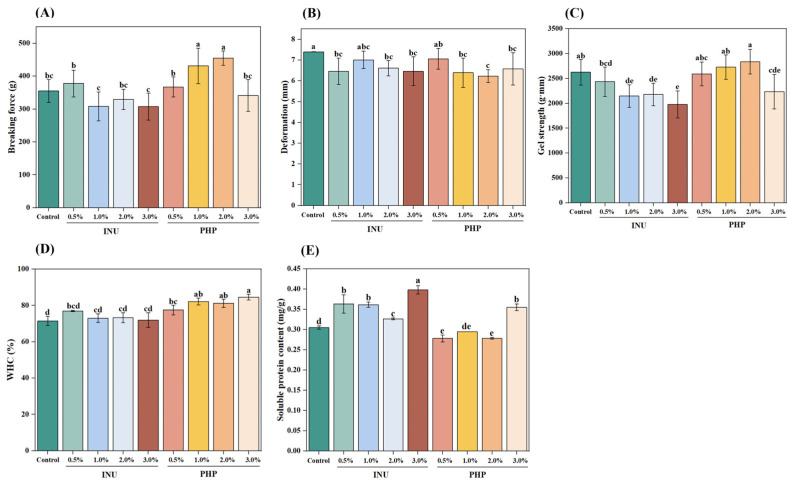
Breaking force (**A**), deformation (**B**), gel strength (**C**), WHC (**D**) and protein solubility (**E**) of blended surimi gels added with different dietary fiber at various concentrations. Note: INU, inulin; PHP, psyllium husk powder. Different lowercase letters on the bars indicate significant differences (*p* < 0.05). Vertical bars represent the standard deviation (n = 3).

In Figure 1A–C, regardless of INU content, the gel strength of INU-added gels was lower than that of the control (2628.03 g·mm) (*p* < 0.05). After 3% INU was added, the gel strength decreased to the minimum (1979.90 g·mm). Notably, the gel strength of the sample with 0.5% INU added decreased to 2434.87 g·mm, whereas the breaking force rose by 6.3%. This enhancement in breaking force may be attributed to the hydroxyl groups present in INU, which substantially promote hydrogen bonding [27]. Besides the disruption of the protein matrix within the gel network caused by INU particles, reduced protein content led to weaker gels [28]. Conversely, incorporating PHP into blended gels increased gel strength (*p* < 0.05). At a dosage of 2% PHP, the gel strength reached a maximum of 2838.60 ± 248.11 g·mm. However, when 3% PHP was added, the gel strength significantly decreased to 1979.90 g·mm (*p* < 0.05). This suggests that increased gel strength was more likely due to the high viscosity and gelatinous properties of PHP, which can damage the structural stability of the gel network and reduce the molecular forces between the protein molecules [29]. Nonetheless, excessive amounts of PHP impeded protein cross-linking and consequently lowered gel strength [9]. Based on a previous study on the effect of adding PHP on the MP gelation of pork loin muscle, 3% PHP could destroy the MP gel network, because the excess PHP filling into the MP gel network blocked the cross-linking of protein molecules [25].

### 3.3. WHC

The WHC of gels reflects the capacity of the protein network to bind or take up water, which closely correlates with textural properties. Typically, a dense and ordered gel network provides sites for water retention and constrains the mobility of water molecules within its structure [24]. The WHC results were altered by incorporating INU or PHP (Figure 1D). When blended gels were supplemented with 0.5% INU (76.93 ± 0.28) or 2.0% PHP (81.22 ± 2.34), WHC values increased. However, the WHC values gradually decreased with the increasing concentration of INU. In contrast, as PHP concentration increased, the WHC fluctuated. INU is regarded as a prebiotic polysaccharide that retains moisture molecules through weak hydrogen bonding (hydroxyl hydration), thereby improving the WHC of gels [8,30]. During the heating process, the hydrophobic groups within the MP are exposed; PHP promotes the formation of a dense protein–polysaccharide network structure through ionic bonds, hydrogen bonds, and hydrophobic interactions. This process eliminates the interconnected water channels and enhances water retention, ultimately improving the WHC results [9,10,31].

### 3.4. Protein Solubility

Non-covalent interactions can influence the conformation of proteins; therefore, protein solubility is used to evaluate protein–protein interactions. Thus, it reflects the gel characteristics [32]. The protein solubility of the gels containing added INU first decreased and then increased with increasing INU (Figure 1E). Conversely, the protein solubility of the samples containing added PHP fluctuated with increasing PHP content. The protein solubility was significantly higher in the gel with 3% added INU than the others (*p* < 0.05). Lower protein solubility was observed in gels with 0.5% and 2% PHP added. Adding PHP reduced the solubility of the gels, suggesting that PHP might promote the formation of non-disulfide covalent bonds [33]. In the present study, a higher protein solubility of a gel indicated a lower formation rate of non-disulfide covalent bonds. Non-disulfide covalent bonds were reduced, and myosin heavy chain cross-linking was inhibited [34,35]. Conversely, the lower protein solubility was related to the increased formation of non-disulfide covalent bonds and improved the gel strength of the surimi [36].

### 3.5. Color

The effects of dietary fiber on the color parameters of blended gels are presented in Table 2. Compared to the control, incorporating INU significantly enhanced the samples’ L*, a*, and whiteness values (*p* < 0.05). This enhancement may be attributed to the denser gel network filled with dissolved INU, which results in a smoother cross-section that facilitates light scattering. Consequently, increased reflected light contributes to improved whiteness [37]. Elevated L*-value and whiteness indicate of superior quality surimi gels [38]. Conversely, adding PHP did not significantly influence the L* and a* values (*p* > 0.05). However, higher concentrations of PHP markedly elevated the b* values (*p* < 0.05). Furthermore, as the amount of PHP increased, the whiteness decreased correspondingly. This phenomenon may be attributed to the inherent faint yellow coloration of PHP, which is in agreement with the observation that high concentrations (>1%) of PHP diminished the whiteness of surimi gels [39].

### 3.6. SEM

**Figure 2:**
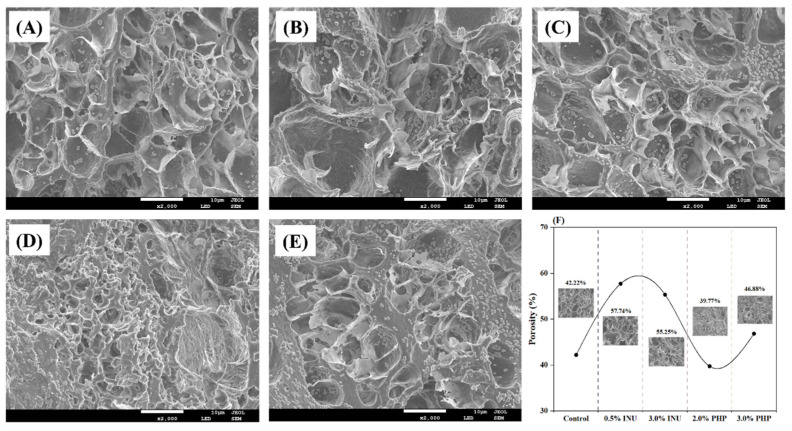
Microstructure images (**A**–**E**) and porosity (**F**) of blended surimi gels added with different dietary fibers at varying concentrations. Note: INU, inulin; PHP, psyllium husk powder. (**A**), Control (without dietary fiber); (**B**), 0.5% INU; (**C**), 3% INU; (**D**), 2% PHP; (**E**), 3% PHP.

In Figure 2, the control has coarse networks with large protein aggregates characterized by numerous voids or cavities within the gel network. This observation is consistent with the control having the lowest WHC, as shown in Figure 1D. Upon adding 0.5–3.0% INU, the gel structure became increasingly disorganized and had larger pores, which supported the observed reductions in TPA and gel strength when INU was incorporated. When PHP was incorporated at a concentration of 2.0%, a dense network with minimal porosity developed within the gel matrix. Simultaneously, the structural integrity of the gel was stable while maintaining structural integrity and exhibiting maximum hardness. PHP fibers contain multiple hydrophilic functional groups that might effectively absorb and stabilize water within the gel matrix, thereby improving its confined water ability. This process inhibited fixed water conversion [40]. However, when the PHP content reached 3.0%, a loosening and roughening of the gel structure occurred. The excessive self-aggregation of dietary fiber presented in PHP could impede the proper formation of a protein network. Additionally, more PHP solid particles likely extended molecular distances between protein chains, thereby interfering with the protein–protein interactions within the gel matrix [9]. Furthermore, adding 0.5% or 3.0% INU increased the porosity of gels by 57.74% and 55.25%, respectively. In contrast, compared with the control (42.22%), incorporating 2% PHP reduced porosity to 39.77%. Notably, by adding 3% PHP to the blended gels, the porosity reached 46.88%. These findings suggest that adding PHP promoted the cross-linking of the gel matrix, which contributed to densifying the three-dimensional network of gels [41].

### 3.7. In Vitro Protein Digestion for Optimized Blended Gels

#### 3.7.1. Protein Digestibility

**Figure 3:**
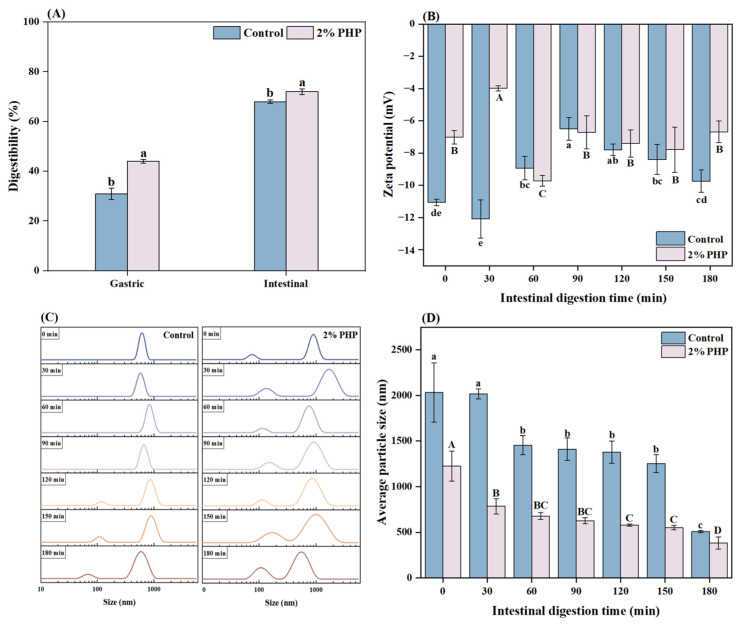
Digestibility (**A**), zeta potential (**B**), particle size distribution (**C**), and average particle size (**D**) of blended surimi gels without and with 2% PHP addition in simulated gastric and intestinal digestion. Note: PHP, psyllium husk powder. Lowercase letters indicate significant differences (*p* < 0.05) in the control group during digestion. Uppercase letters indicate significant differences (*p* < 0.05) in the PHP group during digestion. Vertical bars represent the standard deviation (n = 3).

Adding 2% PHP significantly enhanced the gastric and intestinal protein digestibility of blended gels compared to the control (*p* < 0.05) (Figure 3A). Compared with the control, gastric and intestinal digestibilities for the gel containing 2% added PHP increased by 12% and 3%, respectively. These findings suggest that complexes formed between the polysaccharides and the MP, reducing aggregation and enhancing solubility [42]. Furthermore, the gastrointestinal digestibility of the gels improved with PHP supplementation; this might be attributed to functional components, such as xylose and arabinosaccharide, present in the PHP polysaccharides that likely modify the secondary structure of muscle protein. This could compromise the structural stability of the gel while expediting its digestibility [43]. PHP contains more than 80% dietary fiber, of which 70% is soluble dietary fiber [10]. A high-fiber diet has decreased average retention time in small and large intestines while prolonging stomach retention. This results in an earlier sensation of satiety within the stomach [44]. One strategic approach for reducing energy in food involves enhancing the fiber content. Consequently, increasing dietary fiber via PHP addition accelerates digestibility, potentially positively influencing cardiovascular health, diabetes management, weight control, and immune function [45].

#### 3.7.2. Zeta Potential and Particle Size

The zeta potential signifies the repulsive forces between particles and indicates the stability [46]. The zeta potential observed during in vitro digestion simulations of intestinal samples is shown in Figure 3B. The control and the sample containing added 2% PHP had negative zeta potentials because of the release of anionic bile salts, free fatty acids, and peptides within the small intestine [47]. In the control, there was a significant increase in negative charge from −11.06 ± 0.20 to −12.09 ± 1.19 mV at 30 min (*p* < 0.05), followed by a decrease to −6.49 ± 0.70 mV at 90 min, following extended intestinal digestion. Conversely, the zeta potential of the 2% PHP added gel significantly decreased from −7.02 ± 0.42 to −3.98 ± 0.16 mV at 30 min (*p* < 0.05) and then rose significantly to −9.72 ± 0.33 mV at 90 min (*p* < 0.05). Subsequently, the zeta potential declined as the digestion time increased. During the intestinal digestion phase, the release of bile salt–induced competitive adsorption with interfacial phospholipids, generating a phospholipid–bile salt complex interface. Because bile salt possesses high negative charges within intestinal fluid environments, it more likely contributed substantially to reduced zeta potentials [48].

**Figure 4:**
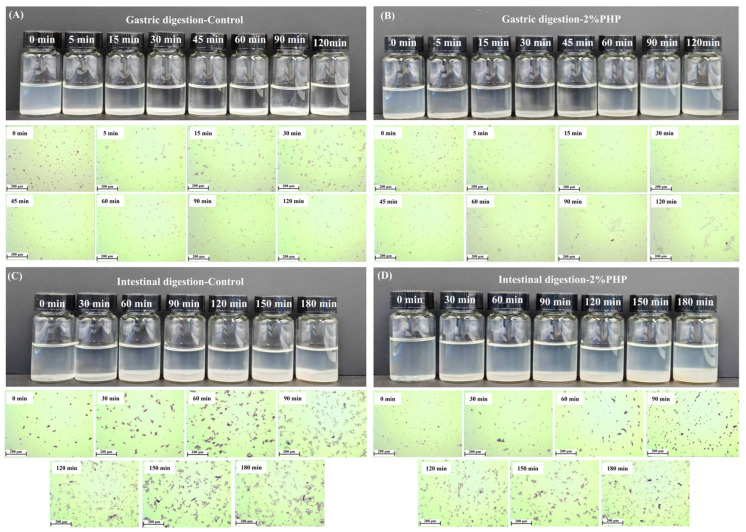
Visual appearance and optical microscopic observation of control gel (**A**,**C**), 2% PHP added gel (**B**,**D**) in gastric and intestinal digestion, respectively. Note: PHP, psyllium husk powder.

The particle size was used to evaluate protein alterations following gastrointestinal digestion [49]. The distribution and mean value of the particle size of the control and the 2% PHP added gel during the in vitro simulated intestinal digestion process are illustrated in Figure 3C,D. Generally, a single peak signifies a monodisperse distribution. In contrast, a narrower peak reflects an improved polydispersity index [50]. At the onset of intestinal digestion, the sample showed a unimodal distribution characterized by a narrow range. As the intestinal digestion progressed, there was a gentle shift in the particle size distribution, indicating a consistent reduction in particle size in agreement with microstructural changes (Figure 4). Throughout the simulated intestinal digestion phase, both groups experienced continuous decreases in particle sizes, reaching their minimum values at 180 min (507.90 ± 11.99 for the control and 382.97 ± 67.05 for the PHP-added gel). This alteration in particle size after digestion could be attributed to pepsin and trypsin, which hydrolyzed proteins into smaller peptides and amino acids [51]. Moreover, during simulated intestinal digestion, all measured particle sizes within the PHP group were significantly smaller than those observed in the control group (*p* < 0.05), suggesting that incorporating PHP effectively enhances the digestibility of blended gels, as depicted in Figure 3A.

#### 3.7.3. Visual Appearance and Optical Microscopy

The visual appearance of the digestive products is presented in Figure 4. As the duration of gastrointestinal digestion progressed, an accumulation of protein particles was observed at the bottom of the digestive fluid. This digestion process was accompanied by changes in pH value, ionic strength, and enzyme activity, which altered the electrostatic repulsion between the protein molecules. These modifications led to peptide bond cleavage and weakened the structure, gradually disrupting the integrated condensates into loose and fragmented structures [52].

Before the simulated digestion, both the control and gel with 2% added PHP exhibited a granular morphology, and the samples changed when they were subjected to pepsin digestion. In the control, macroscopic aggregation was noted after 5 min of gastric digestion, and an aggregation peak occurred at 45 min (Figure 4A). For the gels containing added PHP, the aggregation commenced at 60 min and reached its maximum volume at 120 min (Figure 4B). The accumulation of aggregation may be attributed to the mucins present in the artificial saliva promoting bridge flocculation among MP particles, while pepsin facilitated both digestion and displacement of coatings adsorbed on particle surfaces following simulated gastric digestion [53]. In contrast to these findings, during intestinal digestion, both groups displayed a granular state at 0 min and 60 min, respectively (Figure 4C,D). The proteins were progressively separated from the porous agglomerates and degraded markedly as intestinal digestion time increased. At the end of the intestine phase (180 min), significant degradation of protein particles was evident when compared with their initial state prior to intestinal digestion [54].

#### 3.7.4. Total Sulfhydryl Group Content (TSC)

**Figure 5:**
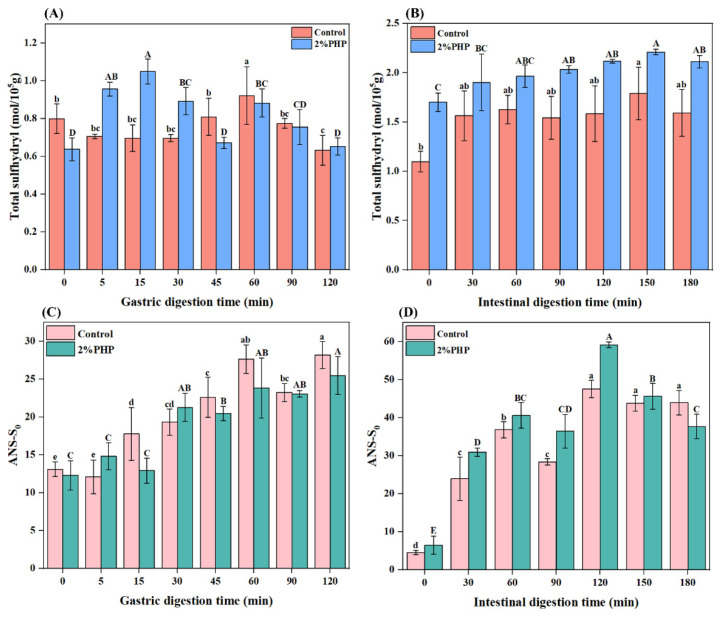
Total sulfhydryl and surface hydrophobicity of control gel (**A**), 2% PHP added gel (**C**) in gastric digestion; total sulfhydryl and surface hydrophobicity of control gel (**B**), 2% PHP added gel (**D**) in intestinal digestion. Note: PHP, psyllium husk powder. Lowercase letters indicate significant differences (*p* < 0.05) in the control group during digestion. Uppercase letters indicate significant differences (*p* < 0.05) in the PHP added gel during digestion. Vertical bars represent the standard deviation (n = 3).

Sulfhydryl groups play a critical role in maintaining the structural integrity of proteins. As the protein structures unfold, these sulfhydryl groups are increasingly exposed on the protein surface, making them susceptible to oxidation and leading to the formation of disulfide bonds. This process reduces the overall content of sulfhydryl groups [55]. The TSC of digestive products during the simulated gastric digestion phase is demonstrated in Figure 5A. With prolonged gastric digestion time, a distinct trend emerged between the control and the gel containing 2% added PHP. In the control, as digestion time extended from 0 min to 30 min, the TSC decreased from 0.80 ± 0.08 mol/10^5^ g to 0.70 ± 0.02 mol/10^5^ g. However, at 60 min, the content increased to 0.97 ± 0.09 mol/10^5^ g. The TSC at the end of gastric digestion (120 min) was significantly lower than that observed at the beginning (*p* < 0.05). Nevertheless, the TSC of the gel containing 2% added PHP increased from 0.64 ± 0.06 to 1.05 ± 0.07 mol/10^5^ g after 15 min. At the last stage (120 min), the TSC decreased to 0.65 ± 0.05 mol/10^5^ g without exhibiting a significant difference from its initial concentration (*p* > 0.05). The fluctuations within the TSC might impact the mutual aggregation and interactions among protein molecules during gastric digestion. Simultaneously, protein or peptide chains expose previously buried sulfhydryl groups [56].

The TSC of the control group and the 2% added PHP gradually increased with prolonged intestinal digestion time, reaching the optimal values at 150 min (1.79 ± 0.27 and 2.21 ± 0.03 mol/10^5^ g, respectively) (Figure 5B). This elevation may be attributed to the proteases and bile salts that caused the partial unfolding of the protein and the buried sulfhydryl groups that were exposed, increasing the TSC [57]. The unfolding rate of the MP was higher than that of aggregation under digestion conditions, exposing some of the sulfhydryl groups [57]. Furthermore, fish proteins can undergo oxidation following exposure to digestive enzymes within the stomach and intestine. The variations in TSC indicate protein oxidation during digestion [58]. Decreasing TSC signifies that the free sulfhydryl groups are either oxidized or engaged in other chemical reactions, resulting in a diminished quantity of available free sulfhydryl groups. This alteration could unfavorably affect the nutritional worth, digestibility, and bioavailability of proteins or peptides [58]. Incorporating PHP into blended gels with a higher TSC might mitigate the oxidative processes related to higher aggregation during gastrointestinal digestion. This could enhance the availability of free sulfhydryl groups, improving both nutritional value and bioavailability.

#### 3.7.5. Surface Hydrophobicity

As the gastric digestion time was extended, the surface hydrophobicity of the control group and the 2% added PHP gel progressively increased, achieving maxima of 28.17 ± 1.74 and 25.47 ± 2.50 at 120 min, respectively (Figure 5C). This enhanced surface hydrophobicity indicates that hydrophobic groups, including amino acid residues (i.e., tryptophan and lysine), were gradually exposed on the molecular surface during digestion [59]. After 60 min of digestion, the surface hydrophobicity of both groups stabilized, suggesting that the protein degradation did not further increase after 60 min, and the exposure of previously concealed hydrophobic regions was reduced [60].

The surface hydrophobicity of simulated intestinal digestive products is illustrated in Figure 5D. Following gastric digestion, pepsin-mediated degradation led to protein dissociation and unfolding and resulted in the exposure of certain hydrophobic groups. As the digestion time was extended, hydrophobic groups aggregated to form new complexes, decreasing surface hydrophobicity. Throughout the prolonged phase of intestinal digestion, both the control and the 2% added PHP gel had an initial increase in surface hydrophobicity followed by a subsequent decrease. Notably, their values reached significant peaks at 120 min. The maximum values of these two samples were 47.53 ± 2.25 (control) and 59.17 ± 0.76 (2% PHP). Because of enzymatic hydrolysis, the rate of exposure of hydrophobic groups surpassed that of aggregate formation between proteins or peptides [61]. However, as protein degradation continued and the substrate concentration decreased, a pronounced reduction in hydrophobicity was observed between 120 and 150 min due to the formation of insoluble aggregates. During simulated intestinal digestion, the surface hydrophobicity of the gel with added PHP consistently exceeded that of the control. This indicates that gels supplemented with PHP were more prone to conformational changes during digestion [62], which corresponded with enhanced digestibility as presented in Figure 3A.

#### 3.7.6. Endogenous Fluorescence Spectrum

**Figure 6:**
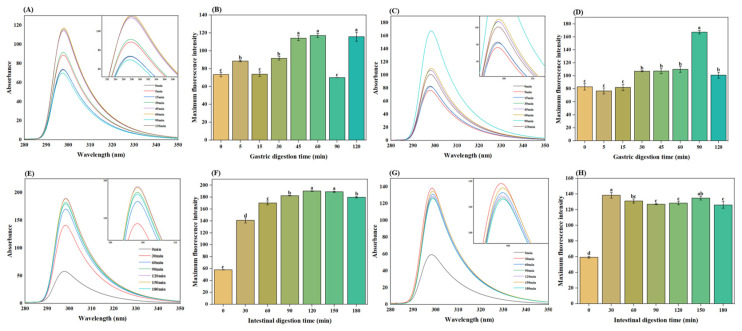
Gastric digestion endogenous fluorescence of the control gel (**A**) and 2% PHP added gel (**C**); gastric digestion fluorescence intensities of control gel (**B**) and 2% PHP added gel (**D**). Intestinal digestion endogenous fluorescence of the control gel (**E**) and 2% PHP added gel (**G**); intestinal digestion fluorescence intensities of control gel (**F**) and 2% PHP added gel (**H**). Note: PHP, psyllium husk powder. Different lowercase letters indicate significant differences (*p* < 0.05) among different groups. Vertical bars represent the standard deviation (n = 3).

The fluorescence patterns and maximum fluorescence intensities of gastric digestive products are presented in Figure 6. In the control, the fluorescence intensity increased with digestion time, except at 15 and 90 min. The highest fluorescence was observed at 60 min with a value of 117.20 ± 2.26. Similarly, the gel containing added PHP exhibited an analogous increasing trend and achieved its maximum (167.23 ± 2.80) at 90 min. The decrease in fluorescence intensity indicated that peptide bonds were cleaved during digestion, characteristic groups were exposed, and intermolecular forces were enhanced [63]. Simultaneously, the hydrophobicity of the microenvironment where the tryptophan residues are located increased, and the number of hydrophobic functional groups within the protein increased. This outcome agrees with the findings of the hydrophobicity assay [63]. High fluorescence intensity indicates the presence of hydrophobic amino acid residues, such as tryptophan residues, in a folded state [64].

As depicted in Figure 6E,G and Figure 6F,H, both the control and gel containing added PHP exhibited an accumulation in fluorescence intensity accompanied by a blue shift. Peak values were recorded at 120 min for the control (190.23 ± 0.52) and at 30 min for the PHP-added gel (138.37 ± 3.44). This enhancement may be attributed to trypsin, in which tryptophan embedded in hydrophobic regions became exposed during intestinal digestion [65]. The observed blue shift likely resulted from fluorophores, such as Trp and Tyr, sequestered into a more non-polar environment due to conformational changes induced during intestinal digestion [66]. The intrinsic fluorescence intensity gradually increased throughout digestive processing. Proteases continuously hydrolyzed the surimi protein into peptides, leading to greater exposure of tryptophan residues [67], which also reflected the improved digestibility of the surimi.

## 4. Conclusions

Incorporating INU adversely affected gel properties, as evidenced by decreased textural properties and gel strength. In contrast, adding 2.0% PHP significantly enhanced two indicators and reduced the porosity of the gel matrix. Furthermore, incorporating PHP markedly increased the digestibility, TSC, and surface hydrophobicity of blended gels. Synchronously, adding PHP significantly reduced the particle size of the digestible products (*p* < 0.05). Therefore, PHP effectively enhanced both the gel performance and its digestibility. This study provides valuable theoretical insights and guidance for producing high-nutritional-value surimi-based products with high digestibility.

## Figures and Tables

**Table 1:** Effect of INU or PHP at different concentrations on textural properties of blended surimi gels.

Sample		Hardness (N)	Cohesiveness	Springiness	Chewiness (mJ)
Control		45.33 ± 2.66 bc	0.50 ± 0.03 a	5.42 ± 0.43 a	122.11 ± 10.70 abc
INU	0.5%	44.63 ± 0.84 bc	0.44 ± 0.02 bc	5.38 ± 0.46 a	105.40 ± 6.88 bcd
	1%	44.93 ± 3.04 bc	0.43 ± 0.02 bc	5.33 ± 0.53 a	104.59 ± 16.78 bcd
	2%	44.43 ± 0.62 bc	0.41 ± 0.04 bc	5.24 ± 0.40 a	97.81 ± 16.45 cd
	3%	40.87 ± 2.85 c	0.39 ± 0.01 c	5.12 ± 0.44 a	82.11 ± 10.70 d
PHP	0.5%	48.77 ± 4.17 b	0.44 ± 0.01 bc	5.45 ± 0.34 a	117.37 ± 15.87 abc
	1%	51.23 ± 5.07 ab	0.47 ± 0.02 ab	5.61 ± 0.21 a	133.90 ± 13.86 ab
	2%	56.43 ± 4.75 a	0.46 ± 0.01 ab	5.68 ± 0.34 a	146.71 ± 8.91 a
	3%	47.1 ± 2.38 bc	0.39 ± 0.03 c	5.51 ± 0.40 a	100.39 ± 17.00 cd

Note: INU, inulin; PHP, psyllium husk powder. Values are presented as mean ± SD (n = 3). Different lowercase letters in the same column indicate significant differences (*p* < 0.05).

**Table 2:** Effect of different INU or PHP at different concentrations on color of blended surimi gels.

Sample		L*	a*	b*	Whiteness
Control		82.31 ± 0.56 bc	8.11 ± 0.17 bcd	13.13 ± 0.25 bc	76.52 ± 0.61 bc
INU	0.5%	84.58 ± 1.12 a	8.45 ± 0.12 a	12.50 ± 0.33 cd	78.42 ± 0.82 a
	1%	84.47 ± 1.41 a	8.33 ± 0.05 ab	12.26 ± 0.14 d	78.52 ± 1.10 a
	2%	83.25 ± 0.17 ab	8.13 ± 0.07 bcd	12.50 ± 0.40 cd	77.58 ± 0.37 ab
	3%	83.02 ± 0.73 ab	8.36 ± 0.04 ab	13.09 ± 0.08 bc	76.99 ± 0.50 abc
PHP	0.5%	83.32 ± 0.20 ab	8.00 ± 0.03 cd	12.59 ± 0.26 bcd	77.62 ± 0.31 ab
	1%	82.37 ± 0.33 bc	7.89 ± 0.03 d	12.61 ± 0.31 bcd	76.93 ± 0.41 abc
	2%	80.57 ± 0.22 c	8.22 ± 0.10 abc	13.93 ± 0.31 a	74.72 ± 0.15 d
	3%	81.45 ± 0.86 bc	7.89 ± 0.09 d	13.31 ± 0.32 ab	75.84 ± 0.85 cd

Note: INU, inulin; PHP, psyllium husk powder. Values are presented as mean ± SD (n = 3). Different lowercase letters in the same column indicate significant differences (*p* < 0.05).

## Data Availability

The original contributions presented in the study are included in the article, further inquiries can be directed to the corresponding authors.
